# Chemical Composition, Antioxidant, and Antitumor Activity of Fucoidan from the Brown Alga *Dictyota dichotoma*

**DOI:** 10.3390/molecules28207175

**Published:** 2023-10-19

**Authors:** Mostafa M. El-Sheekh, Fatma Ward, Mohamed A. Deyab, Majid Al-Zahrani, Hussein E. Touliabah

**Affiliations:** 1 Department of Botany, Faculty of Science, Tanta University, Tanta 31527, Egypt; mostafaelsheikh@science.tanta.edu.eg; 2 Department of Botany and Microbiology, Faculty of Science, Damietta University, New Damietta City 34511, Egypt; 3 Department of Biological Science, College of Science and Arts at Rabigh, King Abdulaziz University, Rabigh 25732, Saudi Arabia; maalzahrani4@kau.edu.sa; 4 Faculty of Women for Ats, Science and Education, Ain Shams University, Cairo 11757, Egypt

**Keywords:** *Dictyota dichotoma*, antioxidant activity, antitumor activity, Fucoidan with sulphated polysaccharides, Phaeophyta, Red Sea

## Abstract

Brown macroalgae are a rich source of fucoidans with many pharmacological uses. This research aimed to isolate and characterize fucoidan from *Dictyota dichotoma* var. *dichotoma* (Hudson) J.V. Lamouroux and evaluate *in vitro* its antioxidant and antitumor potential. The fucoidan yield was 0.057 g/g algal dry wt with a molecular weight of about 48.6 kDa. In terms of fucoidan composition, the sulfate, uronic acid, and protein contents were 83.3 ± 5.20 mg/g fucoidan, 22.5 ± 0.80 mg/g fucoidan, and 26.1 ± 1.70 mg/g fucoidan, respectively. Fucose was the primary sugar component, as were glucose, galactose, mannose, xylose, and glucuronic acid. Fucoidan exhibited strong antioxidant potential that increased by more than 3 times with the increase in concentration from 0.1 to 5.0 mg/mL. Moreover, different concentrations of fucoidan (0.05–1 mg/mL) showed their ability to decrease the viability of Ehrlich ascites carcinoma cells in a time-dependent manner. These findings provided a fast method to obtain an appreciable amount of natural fucoidan with established structural characteristics as a promising compound with pronounced antioxidant and anticancer activity.

## 1. Introduction

The Red Sea is a rich and highly productive ecosystem for marine organisms due to the unique coral reefs extending along the coastline and water temperature fluctuations. The Red Sea is a favorite aqua system for bioactive macroalgal growth along Egypt’s coasts. Approximately 500 seaweed species have been recorded in the Red Sea. Marine macroalgae gain significant importance due to their content of potent bioactive substances such as polysaccharides, fatty acids, heterocyclic carbons, alkaloids, cyclic peptides, and amino acids [1,2]. Brown algae are the largest group of algae, including 1500–2000 species, and are mostly marine and macroscopic. *Dictyota dichotoma* is a brown seaweed belonging to the family Dictyotaceae and the order Dictyotales. *D. dichotoma* has a flattened, dichotomously branched thallus without midribs and veins and is anchored to the substrate by rhizoids that terminate as an adhesive disc. *D. dichotoma* represents one of the most important algae in coral reef ecosystems [3].

In brown algae, Fucoidan, laminarin, alginate, and mannitol are the major storage carbon compounds that exhibit many novel physiological, biological, metabolic, and ecological characteristics. The reproductive phase and collection site play an important role in the content of these active principles and the biological activity of brown seaweed *in vitro* anti-inflammatory activities of fucoidans from five species of brown seaweeds [4]. Fucoidan is a sulfated polysaccharide extracted from brown algal cell walls such as those of *Dictyota menstrualis*, *Padina boryana*, *Kjellmaniella crassifolia*, and *Fucus vesiculosus* [5]. The extraction of fucoidan from some brown macroalgae involves multi-extractions, usually by using hot hydrochloric acid, and perhaps includes adding calcium to precipitate alginate during purification [6]. Still, no standardized extraction method is known for fucoidans at present. The extraction method significantly influences the yield and composition of algal polysaccharides [7].

Species of algae and their habitats greatly affect the structure of fucoidan and its chemical composition. Moreover, extraction methods significantly affect the structure and bioactivity of fucoidan [8,9,10,11]. Its bioactivity is due to its molecular characteristics, such as sugar types, sulfate content, linkages, and molecular geometry. Fucoidan could be identified based on its molecular weight by dividing it into low and high molecular weights. Specific *Dictyota* metabolites, such as fucoidans or polyphenols revealed antiviral, anti-nociceptive, antitumor, and anti-inflammatory activities [7,11]. Additionally, fucoidans obtained from *Cystoseira crinite*, *Laminaria hyperborea*, *Fucus*, *F. evanescens*, *F. vesiculosus*, *F. distichus*, and *Ascophyllum nodosum* showed antioxidant, antiviral, anti-inflammatory, anti-hyperglycemic, anti-coagulant, antidiabetic, anticancer, antiradical, and antibacterial activities [12,13,14,15,16,17,18,19]. Moreover, sulfated polysaccharides extracted from *D. dichotoma* var. *velutricata*, *Dictyopteris polypodioides*, and *Turbinaria ornata* showed excellent antioxidant activity [20,21].

Exposure to environmental dangers such as shortwave radiation, contamination, smoking, and herbicides can enhance the generation of free radicals that can destroy DNA, lipids, and essential proteins and cause various human disorders such as cancer, liver injury, and rheumatism. These disorders arise due to “oxidative stress”, which is a difference between the oxidant and antioxidant activities of the body. Antioxidants delay the autoxidation of cellular compartments by reducing the free radical’s formation or interrupting the propagation of the free radical chain with scavenging species and chelating metal ions, thus avoiding peroxide formation and/or reducing oxygen levels. Therefore, the antioxidant ability to scavenge free radicals may greatly prevent and remedy diseases arising from oxidants or free radicals. Hence, antioxidants are vital in diseases such as cancer, aging, inflammation, and Alzheimer’s [22,23]. Brown macroalgae are an excellent source of antioxidants due to their biodiversity and large amounts of various antioxidant compounds. Powerful antioxidant compounds in brown seaweeds include proteins, some pigments (chlorophyll and carotenoids), alkaloids, some vitamins (E and C), glutathione, sulfated polysaccharides, amino acids, amines, and phenolics as flavonoids and coumarins [24,25,26].

Cancer is a major disease burden worldwide and can arise in many body parts. Chemotherapy treats many types of cancer effectively. But like other chemical treatments, it often causes side effects, such as headaches, muscle pain, hair loss, and stomach pain. Marine bioactive products with novel structures can treat some diseases. Till now, cancer treatments do not have safe medicine as the currently available drugs are causing side effects such as vomiting, diarrhea, fatigue, and nausea. Hence, exploring and identifying new safe, cheap, and less toxic anticancer agents from natural sources is important. Nowadays, most pharmaceutical products are derived from various microorganisms, herbal plants, and seaweeds. Fucoidan is a non-toxic compound with therapeutic potential for some diseases. Kim and his colleague reported that fucoidan is used to treat cancer by directly activating different pathways of apoptosis [27]. Our study aimed to find new medications regarding the low side effects of natural marine compounds. Therefore, this work is the first aimed at isolating and characterizing fucoidan from *D. dichotoma* var. *dichotoma* (Hudson) J.V. Lamouroux and evaluating the extracted fucoidan *in vitro* antioxidant and antitumor activity.

## 2. Results

### 2.1. Structure and Chemical Composition of Isolated Fucoidan

**Figure 1:**
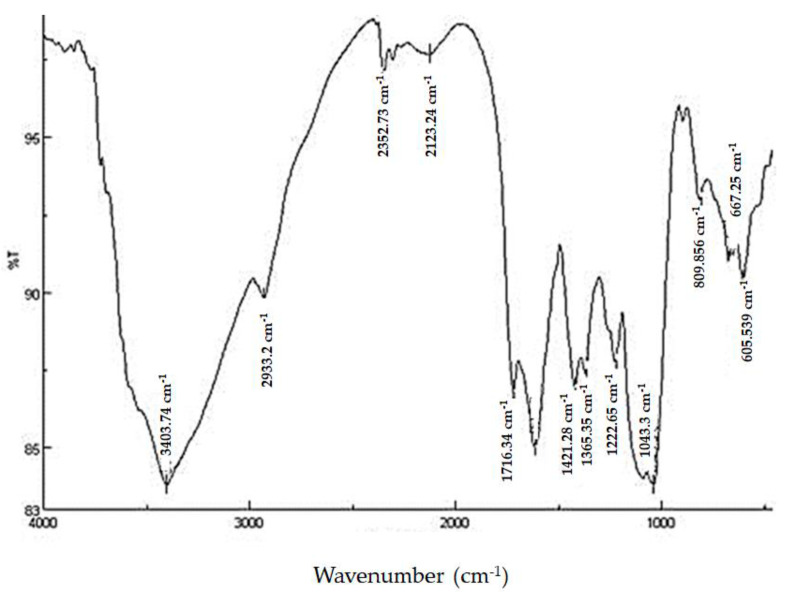
FTIR spectrum of purified fucoidan extracted from *D. dichotoma* var. *dichotoma* (Hudson) J.V. Lamouroux collected from Hurghada shores along the Red Sea Coast of Egypt.

In the current study, the functional groups of the extracted fucoidan powder from *D. dichotoma* var. *dichotoma* were analyzed by FTIR spectroscopy, and the spectrum is given in Figure 1. Lyophilized fucoidan powder showed bands in the regions of 1043.3 and 1222.65, confirming that it is an acidic polysaccharide. The stretching of O–C–O vibration (asymmetric carboxylate) was indicated by asymmetric stretching of carboxylate vibration at 1612.2 and 1716.34 cm^−1^. The bands at 1421.28 and 1365.35 cm^−1^ were assigned to C–OH with a contribution of O–C–O symmetric stretching vibration of the carboxylate group. One of the most important bands was found at 1043.3 cm^−1^, indicating D-glucose, and 1222.65 cm^−1,^ corresponding to ester sulfate groups.

**Figure 2:**
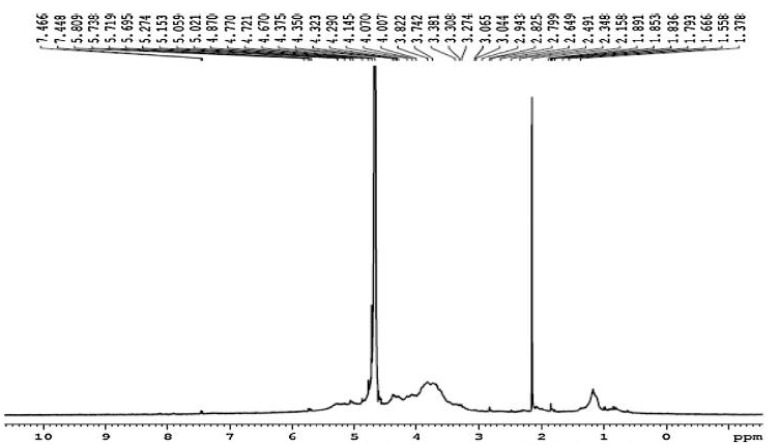
^1^H NMR spectra of purified fucoidan extracted from *D. dichotoma* var. *dichotoma* (Hudson) J.V. Lamouroux collected Hurghada shores along the Red Sea Coast of Egypt.

**Figure 3:**
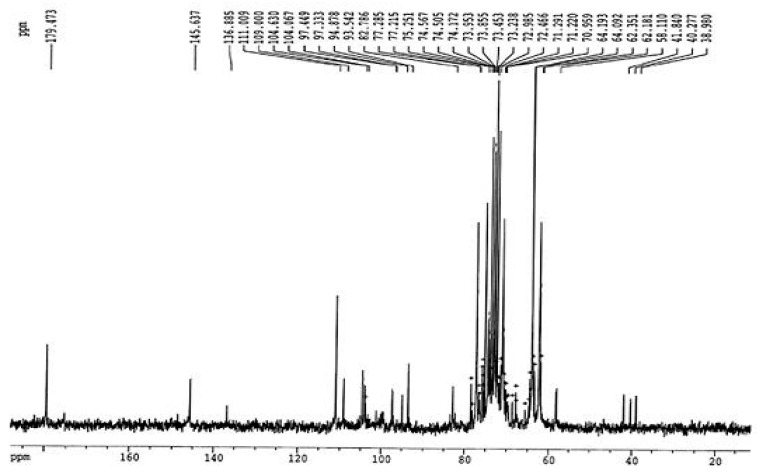
^13^C NMR spectra of purified fucoidan extracted from *D. dichotoma* var. *dichotoma* (Hudson) J.V. Lamouroux collected Hurghada shores along the Red Sea Coast of Egypt.

**Figure 4:**
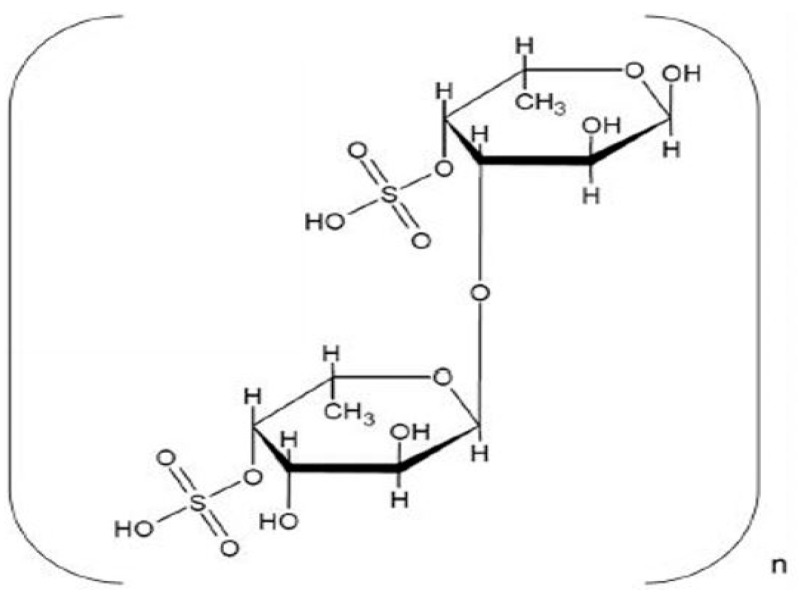
Probable structure of fucoidan extracted from *D. dichotoma* var. *dichotoma* (Hudson) J.V. Lamouroux collected Hurghada shores along the Red Sea Coast of Egypt.

The results of ^1^H NMR spectral analysis of the purified fucoidan extracted from *D. dichotoma* var. *dichotoma* are given in Figure 2. ^1^H NMR showed that signals of protons H-1 arising from α-L-fucose residues and uronic acid residues appeared at alpha-anomeric carbon (5.06 ppm) and C2–C4 hydrogen atoms of a carbohydrate. Signals at 5.69 ppm indicated a 3,4 distribution of α-L-fucose. Signals at 4.87, 4.29, and 4.77 ppm confirmed the shifts of hydrogen atom positions at C2, C3, and C5, respectively. The signal at 1.37 ppm confirmed chemical shifts of hydrogen atoms C6. The double peak at 1.37 ppm corresponded to L-fucopyranose containing two methyl protons at C6. In Figure 3, ^13^C NMR showed sharp signals at 38.98 and 179.5 ppm, corresponding to important O-acetyl carbon regions. It confirmed the presence of the acetyl group. Anomeric carbon signals were obtained at 104.1 and 93.54 ppm. Both signals at 82.78 and 77.22 ppm show the presence of C4 with a sulfate moiety and the C3 position, respectively. From FTIR and NMR spectra, the structural characterization of the fucoidan compound obtained from *D. dichotoma* var. *dichotoma* was presented in Figure 4, which showed a repeated α-(1→3)-linkage. C-4 is always substituted with sulfate groups.

In the current study, the mean sulfate and uronic acid content of the fucoidan extracted from *D. dichotoma* var. *dichotoma* were 8.33 ± 0.52% and 2.25 ± 0.08%, respectively. Moreover, the extracted fucoidan contained 2.61 ± 0.17% protein (Table 1).

As shown in Table 1, HPLC analysis revealed the presence of six monosaccharides, including fucose, glucose, galactose, glucuronic acid, mannose, and xylose, in the extracted Fucoidan. Fucose was the main sugar (21.3 ± 1.30%) in fucoidan obtained in this study, followed by glucose (11.9 ± 0.74%). Glucuronic acid comprises 2.09 ± 0.12%, the lowest % in the sugar composition of the extracted Fucoidan.

The fucoidan yield was 0.057 g/g algal dry weight in the present study. In contrast, the average molecular weight measured by gel filtration using dextrans as a standard for fucoidan was 48.6 kDa.

### 2.2. Physical Characteristics of Fucoidan

The pH value of 1% aqueous fucoidan extracted from *D. dichotoma* var. *dichotoma* was 6.5. The solubility of fucoidan from *D. dichotoma* var. *dichotoma* in different solvents at room temperature was determined. The results revealed that fucoidan was highly soluble in water and sulfuric acid with a maximum saturation of 250 and 200 mg/mL, respectively, and partially soluble in hydrochloric acid with a maximum saturation of 50 mg/mL.

#### 2.2.1. Antioxidant Activity of Fucoidan

Total antioxidant capacity, DPPH, H_2_O_2_, ABTS, nitric oxide, and ferrous ion assays were used as easy, rapid, and sensitive methods to determine the scavenging capacity of the extracted fucoidan. This is followed by the calculation of IC_50_ values (with 95% confidence intervals). In the present study, a wide range of fucoidan concentrations (0.1–5.0 mg/mL) were screened for their antioxidant potential compared to standard ascorbic acid (positive control). The results in Table 2 indicate that fucoidan from *D. dichotoma* var. *dichotoma* expressed appreciable antioxidant potential that increased significantly (*p* < 0.05) with increased concentration. The extracted fucoidan showed high total antioxidant capacity compared to standard ascorbic acid, with a maximum activity of 71.76 ± 1.5% at 5.0 mg/mL; and low IC_50_ (1.41 mg/mL). Moreover, fucoidan has strong scavenging activities against DPPH radicals at different concentrations, with an IC_50_ of 4.59 mg/mL. The maximum activity of fucoidan was recorded as 50.51 ± 1.9% at 5.0 mg/mL. In terms of total antioxidant capacity measured by the other assays, the values reported followed a similar trend to that observed for the DPPH scavenging capacity. Different concentrations of fucoidan obtained from *D. dichotoma* var. *dichotoma* exhibited their ability to scavenge hydrogen peroxide, ABTS, and nitric oxide radicals with an IC_50_ of 4.84 mg/mL, 8.55 mg/mL, and 9.30 mg/mL, respectively. The isolated fucoidan exhibited a low ability to chelate ferrous ions with high IC_50_ (61.9 mg/mL). The values of all fucoidan concentrations showed higher scavenging activity than standard ascorbic acid (positive control).

#### 2.2.2. *In Vitro* Antitumor Activity

**Figure 5:**
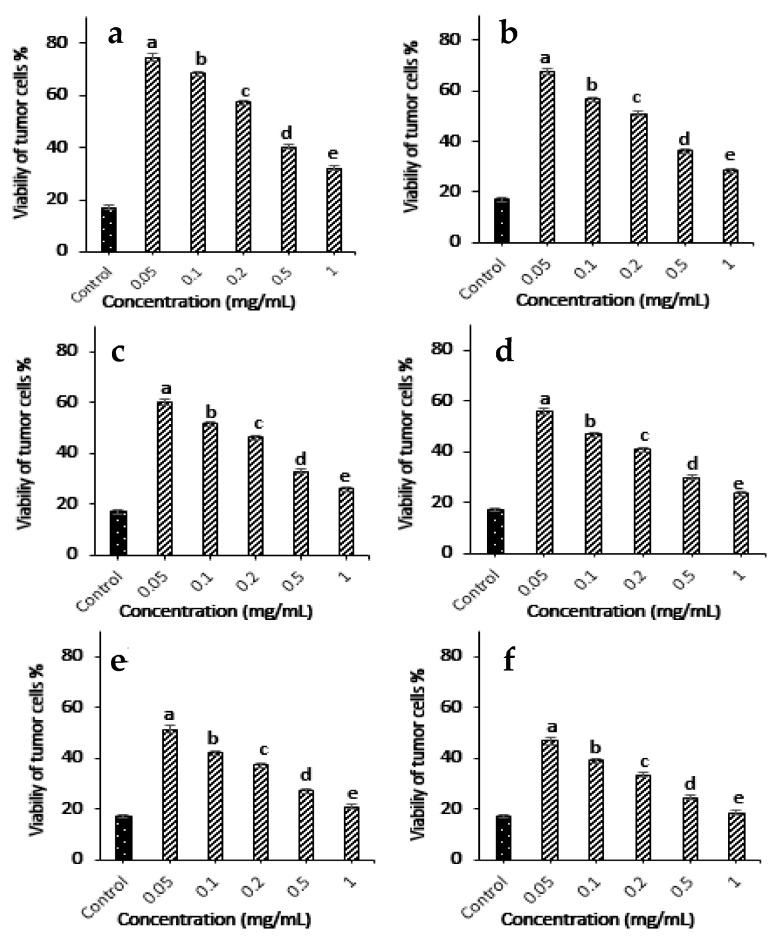
Antitumor activity of different concentrations of the extracted fucoidan at 5 min (**a**), 10 min (**b**), 15 min (**c**), 20 min (**d**), 25 min (**e**) and 30 min (**f**). The control group was normal cells without any treatment.

The present results reveal that different concentrations of fucoidan extracted from *Dictyota dichotoma* var. *dichotoma* in the range (0.05–1 mg/mL) showed their ability to reduce the viability of Ehrlich ascites carcinoma cells, as shown in Figure 5. These results indicated that treatment with different concentrations of fucoidan reduced tumor cell viability significantly in a time-dependent manner (*p* < 0.05). The high concentration (1 mg/mL) of fucoidan can also reduce tumor cell viability to 18.3% after 30 min.

## 3. Discussion

FTIR and NMR spectroscopy are important techniques used to deduce the structure of complex polysaccharides. The broadband at 3403.74 cm^−1^ was assigned to the hydrogen-bonded O–H stretching vibration of the polysaccharide, and that at 2933.2 cm^−1^ corresponds to the C-6 groups of fucose and galactose units. The characteristic absorption at 809.95 cm^−1^ indicated the α-configuration of the sugar units and the characteristic C–O–S stretching of the sulfate group [28]. The occurrence of sulfate groups in the extracted polysaccharide is represented by peaks at 800–900 cm^−1^. The higher sulfate revealed antioxidant activity [29]. The 605.53 and 667.25 cm^−1^ absorption indicated sulfate ester groups and revealed the sulfate group’s symmetric and asymmetric O=S=O deformation [30]. FTIR and NMR spectra in the present study revealed the presence of fucose-sulfated groups linked via repeated α-(1→3)-linkage, and C-4 is always substituted with sulfate groups, whereas the backbone of fucoidan from *Fucus distichus* is made up of alternating 2-sulfated 1,3- and 1,4-linked α-L-fucose residues [31]. NMR and monosaccharide analyses showed that fucose content (21.3 ± 1.30%) is higher than galactose (5.14 ± 0.65%). This confirms that more fucose molecules are present in the side chains and branches of the polysaccharide.

As shown in Figure 4, the FTIR and NMR spectra of the extracted fucoidan exhibit notable differences in absorption patterns to those of fucoidans extracted from *Sargassum cristaefolium*, *Sargassum* sp., *Turbinaria* sp., *Fucus vesiculosus*, and *Padina* sp. [32,33,34,35]. This confirmed that fucoidan composition is influenced by the extraction method and algal species. This difference in the chemical structure of fucoidan is also perhaps because of complex environmental factors such as temperature, light intensity, light day length, and nutrient concentration, in addition to algal changes such as growth, morphology, and reproduction. Moreover, some previous studies reported that the reproductive phase has a significant impact on the biochemical composition and antioxidant properties of fucoidan, as extracted from *Fucus vesiculosus* [15].

The average yield of fucoidan extracted from *D. dichotoma* var. *dichotoma* in the present work (5.7%) was higher than that obtained for *Fucus* spiralis (1.35%), Cystoseira *crinite* (2.80%), *C. sedoides* (3.30%), *Sargassum tenerrimum* (3.60%), *C. compressa* (3.70%), *Fucus versiculosus* (4.00%), and *S. myriocystum* (5.52%) [23,36,37], but lower than the yields reported from *Sargassum linifolium* (13.04%) and *Stypopodium schimperi* (9.09%), *Undaria pinnatifida* (8.8%), *Fucus distichus* (8.69), *Sargassum wightii* (7.15%), and *Sargassum polycystum* (7%) [18,38]. The difference in fucoidan yields was possibly due to the differences in algal species, collection area, degree of maturation, and extraction procedures.

The molecular weight of fucoidan differs according to algal species and environmental conditions. This agrees with Hahn [39] and Kawamoto [40]. They found that the molecular weight of fucoidan ranges from 13 to 627 kDa and may reach 3080 kDa.

Fucoidans extracted from brown macroalgae can be low molecular weight fucoidans, medium molecular weight fucoidans, or high molecular weight fucoidans with a polymer size less than ten kDa, ranging from 10 to 10,000 kDa, or more than 10,000 kDa, respectively [40]. The average molecular weight of fucoidan from *D. dichotoma* var. *dichotoma* was 48.6 kDa (low molecular weight fucoidan). The molecular weight of fucoidan (48.6 kDa) obtained in this study was smaller than those reported for *Undaria pinnatifida* (190 kDa), *Ascophyllum nodosum* (417 kDa), *Saccharina longicruris* (454 kDa), and *Fucus vesiculosus* (735 kDa), and higher than those reported for *Laminaria japonica* (10.5 kDa) and *Saccharina longicruris* (44 kDa) [41,42,43,44,45,46,47,48].

Fucoidan was insoluble in all tested organic solvents in the present study, including ethanol, methanol, acetone, chloroform, diethyl ether, and petroleum ether. These results were confirmed by Hahn [39], who concluded that fucoidan from brown algae is freely soluble in solvents of higher dielectric constants as water due to isolated shielded opposite groups and isolated from other co-extracted natural compounds by solvents of lower dielectric constants as ethanol.

Fucoidan is a sulfated polysaccharide mainly containing fucose, sulfate, monosaccharides, uronic acids, and protein [45]. It was observed that the sulfate content of the fucoidan obtained from *D. dichotoma* var. *dichotoma* was higher than that of fucoidan extracted from *Colpomenia sinuosa* (5.8%) and lower than that found in *Hydroclathrus* (18.1%) and *F. distichus* (38.3%) [31,45]. Yang [33] reports that the bioactivities of fucoidan are strongly related to its sulfate content. The uronic acid content of fucoidan extracted from *Sargassum stenophyllum*, and *Cladosiphon okamuranus* is 3.5% and 23.4%, respectively [38,47]. The fucoidan obtained from *D. dichotoma* var. *dichotoma* contained 2.61 ± 0.17% protein, which was lower than those reported in fucoidan extracted from *Sargassum binderi Sonder* (5.5%), and *S. longicruris* (12.4%) [48]. The difference in protein content was possibly due to the differences in algal species and extraction procedures.

The present study revealed the presence of fucose, glucose, galactose, mannose, xylose, and glucuronic acid in the extracted fucoidan. A similar result has been reported by Dias [49] on fucoidan extracted from *Sargassum stenophyllum*, which had the same six monosaccharides as the main components. Xylose is one of the common minor carbohydrates in brown seaweeds. Fucose, xylose, and their ratios could be used to characterize brown seaweeds as indicators of fucoidan. The ratio of fucose to xylose in fucoidan extracted in the present study (8.38) was lower than that in fucoidan extracted from *F. distichus* (10.42) collected from the western coast of Iturup Island in the summer [37]. It can also provide information about the biological activity of brown seaweeds [5]. Previous reports found that the fucose content of fucoidan obtained from *Padina tetrastromatica*, *Fucus evanescens*, *S. tenerrimum*, *Cystoseira sedoides*, *C*. *compressa*, and *C. crinite* was 54%, 54.9%, 59.3%, 54.5%, 61.5%, and 43.3%, respectively [37], which were more than the content of fucose sugar obtained in this study. Whereas, the fucose:sulfate ratio in fucoidan isolated from *D. dichotoma* in the present study (2.55:1) differs from that isolated from *F. distichus* (1:1.21) [31].

In the present study, different concentrations of fucoidan exhibited considerable antioxidant activity that increased with the increase in concentration. Other researchers agreed with the present study that sulfated polysaccharides obtained from some seaweed and fruits showed dose-dependent reducing powers and free radical scavenging effects.

In the phosphomolybdenum method, Mo (VI) is reduced to form a green phosphate–Mo (V) complex at an acid pH. The scavenging activity percentage of the extracted fucoidan (16.26%) at 0.1 mg/mL was lower than those reported for standard ascorbic acid (84%), and fucoidan obtained from *Sargassum swartzii* (32.34%) [50,51]. DPPH is a stable radical scavenged by an antioxidant compound through hydrogen donation to form a stable yellow DPPH-H molecule. The range of mean values of DPPH (%) in the present study (15.07–51.13%) differs from those reported for algal extracts of *Ulva lactuca* (4.85–76.0%), *Enteromorpha intestinalis* (8.66–76%), and *Cladophora vagabunda* (14.6–78.5%), with a high IC_50_ value (4.59 mg/mL). This DPPH IC_50_ value of the extracted fucoidan was also higher than that of alginate extracted from *Colpomenia sinuosa* (46.2 µg/mL^−1^) [52]. Hence, the effect of the extracted fucoidan on DPPH radical scavenging could be due to their hydrogen-donating ability or may be related to their high sulfate content. A high percentage of DPPH radical scavenging activity was also reported in the methanol extract of brown seaweed, *Turbinaria conoides*, *Polycladia indica*, *Turbinaria ornata*, and *Laurencia obtusa*, and red seaweed, *Sarconema scinaioides* [53].

The ABTS radicals are produced by the reaction of ABTS with H-atom donor antioxidants. These radicals convert into a non-colored form of ABTS, and absorbance decreases. In this study, the maximum activity was found at a concentration of 5 mg/mL to be 50.51%, which was lower than those stated by Tariq [20] for polysaccharides obtained from *D. dichotoma* var. *velutricata* (76.87%) and *Sargassum variegatum* (86.93%). This activity may be related to the fucoidan’s ability to act as a free radical scavenger or by donating a hydrogen atom to the molecule.

Antioxidant compounds can scavenge free radicals, for instance, nitric oxide, by donating protons. This study showed that the extracted fucoidan (at different levels) in sodium nitroprusside solution could decrease the nitrite levels with an IC_50_ of 9.30 mg/mL by their ability to chelate nitric oxide. The reduction in the nitrite level may be due to competition between fucoidan and oxygen in the reaction with NO [54].

Ferrozine can react with Fe^2+^ and form a red ferrozine–Fe^2+^ complex in a ferrous ion chelating assay. Still, in the presence of ion-chelating agents (antioxidant compounds), the complex formation is disrupted, resulting in a decrease in the red color of the complex. The capacity of ferrous ion chelating is expressed by the inhibition percentage of ferrozine–Fe^2+^ complex formation. Fucoidan obtained from *D. dichotoma* var. *dichotoma* exhibits a low chelating ability to ferrous ions with a high IC_50_ (61.9 mg/mL) at diverse concentrations. The ability (11.70 ± 0.6%) of 1 mg/mL fucoidan obtained in this study was lower than those reported in previous studies [54,55,56]. This difference may be due to alterations in algal species and the fucoidan extraction method. The antioxidant activity of the extracted fucoidan may be due to its effect as a ROS scavenger by removing free hydroxyl radicals and superoxide radicals. There are several factors that determine the antioxidant activity of fucoidan, including concentration, molecular weight, degree of sulphation, substitution groups and their positions, type of sugar, and glycosidation branching [57,58,59,60].

Some existing organisms in the marine ecosystem, such as macroalgae, are rich sources of bioactive substances that exhibit antitumor potential in Ehrlich’s ascites carcinoma cells. It was selected in the present study because it is one of the most common experimental tumor models characterized by high translatability, rapid replication, a short lifespan, and no tumor-specific transplantation antigen. It comprises both tumor cells present as single cells, or spheroids, and stromal cells. Moreover, it was easy to obtain in relatively large amounts at a low cost from the peritoneum of mice. Diverse concentrations of the extracted fucoidan from 0.05 to 1 mg/mL showed their ability to decrease the viability of Ehrlich ascites carcinoma cells in a time-dependent manner. The air-dried powder of *Sargassum ringgoldianum*, *Scytosiphon lomentaria*, *Lessonia nigrescens*, as well as *Laminaria japonica* showed significant inhibition against Ehrlich carcinoma cells of 46.5%, 69.8%, 60.0%, and 57.6%, respectively. Wang [57] also reported that fucoidan obtained from *Sargassum thunbergii* inhibits the viability of Ehrlich ascites carcinoma and lung cancer in vivo by inhibiting tumor angiogenesis. Moreover, sulfated polysaccharides from the brown seaweed *Dictyota caribaea* delayed tumor growth in mice bearing sarcoma in vivo without cytotoxic activity against tumor cells *in vitro* [59]. Arumugam [61] reported that fucoidan had more significant anticancer activity compared to quercetin. Previous studies reported that fucoidan has a broad range of impacts on cellular functions, including cell cycle regulation, RNA metabolism, protein metabolism, carbohydrate metabolism, bioenergetics, mitochondrial maintenance, and DNA repair pathways. Induction/inhibition of reactive oxygen species (ROS), mitochondrial instability, and caspase and poly (ADP-ribose) polymerase (PARP) cleavage are all aspects of it [62,63].

## 4. Materials and Methods

### 4.1. Collection of Algae

*Dictyota dichotoma* var. *dichotoma* (Hudson) J.V. Lamouroux was collected at Hurghada, Egypt, on the Red Sea. Its fronds were harvested at the reproductive phase of the vegetatively propagated gametophyte in autumn 2017 and rinsed with seawater to remove epiphytes and debris, then tap and distilled water many times to remove salts. Blotting paper removed surplus water from *D. dichotoma* fronds. The sample was shade-dried and oven-dried at 60 °C for 4 h. The dried algae were pulverized into 2 mm particles. Dr. M. Deyab recognized the alga samples, and the voucher specimen was placed at the Phycology Laboratory (Damietta University, Faculty of Science).

### 4.2. Extraction and Purification of Fucoidan

With few changes to the Yang technique [62], fucoidan was isolated from *D. dichotoma* var. *dichotoma* (Hudson) J.V. Lamouroux fronds. About 30 g of powdered fronds was shaken in a 400 mL solvent system (ethanol:water:formaldehyde) for 24 h at room temperature to remove pigments and polyphenols. Then the mixture was centrifuged at 4000 rpm for 15 min. The supernatant was withdrawn, and the algal residue was washed several times with 400 mL of acetone, followed by centrifugation at 4000 rpm for 10 min. The supernatant was withdrawn, and the residue dried at room temperature, then extracted with 500 mL of 0.2 N HCl for 24 h and filtered through a nylon cloth. Extraction was repeated with 500 mL of 0.2 N HCl at 70 °C for 2 h, followed by 100 mL of distilled water at 65 °C for 2 h. The combined extracts were neutralized with 0.5 N NaOH and filtered through a nylon cloth. The filtrate was incubated with 1% CaCl_2_ in a ratio of 2:1 at 4 °C for 24 h to precipitate alginates. After centrifugation at 4000 rpm for 15 min, the supernatant was incubated overnight with 1 L of 99% ethanol at 4 °C. The precipitate (crude fucoidan) was collected after centrifugation at 6000 rpm for 15 min and spread to dry at room temperature overnight [39,64,65].

### 4.3. Fourier Transforms Infrared (FTIR) Spectroscopy

The extracted powder was identified according to the method described by Kemp [66].

### 4.4. Hydrolysis of Fucoidan

The extracted fucoidan was hydrolyzed by the method described by Nagaoka [67].

### 4.5. Nuclear Magnetic Resonance (NMR)

Three milligrams of purified fucoidan powder were dissolved in 0.5 mL of 99.96% D_2_O (deuterated water) and analyzed by ^1^H and ^13^C NMR on a Bruker Biospin Avance 400 NMR spectrometer (Bruker, Billerica, MA, USA) (^1^H frequency = 400.13 MHz, ^13^C frequency = 100.62 MHz) at 298 °K using a 5 mm inverse broadband probe head with a shielded z-gradient and XWIN-NMR software version 3.5 with TMS as One pulse sequence produced one-dimensional ^1^H and ^13^C spectra. One-dimensional ^13^C spectra utilizing SEFT and QCD 42 sequences were also used to identify the structure.

### 4.6. Molecular Weight Analysis

The molecular weight of pure fucoidan was measured using a size-exclusion HPLC column Superdex 200 (300 mm × 10 mm ID) and a SHIMADZU HPLC system (SHIMADZU HPLC, Kyoto, Japan) with a refractive index detector. The chromatographic eluent was 0.2 M NaCl at 0.3 mL/min. The sample concentration was 10 mg/mL. The injection volume was 0.1 mL at 25 °C. Dextrans with molecular weights of 20, 50, 150, 200, and 670 kDa calibrated the column.

### 4.7. Physical Characteristics of Fucoidan

#### 4.7.1. pH of Fucoidan

The 1% aqueous fucoidan pH was measured using a JENCO 6209 pH meter (JENCO, Scottsdale, AR, USA).

#### 4.7.2. Solubility of Fucoidan

The solubility of fucoidan in different solvents was examined and expressed as mg/mL.

#### 4.7.3. Estimation of Sulfate Content

The extracted fucoidan’s sulfate was estimated turbidimetrically by Dodgson and Price in triplicates [68], and the mean value was reported.

#### 4.7.4. Estimation of Uronic Acid Content

According to Bitter and Muir [69], the uric acid content was determined in triplicates, and the mean value was recorded.

#### 4.7.5. Estimation of Protein Content

Proteins were separated from the other compounds in the extracted fucoidan according to Rajauria et al. [70]. Proteins were precipitated by 100% ethanol overnight at 4 °C and removed by centrifugation. Protein was then determined by the Bradford method using bovine serum albumin as a standard [71]. Protein was added to a 5 mL Coomassie blue dye reagent and mixed well. The optical density was measured at 595 nm against a blank. Protein concentration was determined from a standard curve using bovin in the range of 20–100 µg/mL. Coomassie brilliant blue G-250 was prepared by dissolving 100 mg of the compound in 50 mL of 95% ethanol. To this solution, 100 mL of 85% (*W*/*V*) phosphoric acid was added, then diluted to one litter to give a final concentration of 0.01 (*W*/*V*) coomassie brilliant blue G-250, 4.7% (*W*/*V*) ethanol, and 8.5% (*W*/*V*) phosphoric acid [72].

#### 4.7.6. Analysis of Monosaccharides

Reverse-phase HPLC was used to identify monosaccharide content in fucoidan by the method of Wang [72]. The extracted fucoidan was hydrolyzed into monosaccharides with sulfuric acid (12 M, 0.5 mL) for half an hour. Afterward, the hydrolysate was neutralized, filtered, and subjected to a liquid chromatography (HPLC) step using different reference monosaccharides (mannose, glucosamine, rhamnose, ribose, erythrose, glucuronic acid, galacturonic acid, glucose, galactose, xylose, and fucose). The HPLC column (Bio-Rad, Hercules, CA, USA) was operated at 80 °C and coupled with a refractive index detector. The elution system was 0.1 mol/L aqueous ammonium acetate (solvent B) (pH 4.5) and acetonitrile (solvent A). The injection temperature was maintained at 30 °C. Previous steps were repeated in triplicate, and the mean values were recorded.

#### 4.7.7. Evaluation of the Antioxidant Activity of Fucoidan *In Vitro*

Diverse levels (0.1, 0.5, 1, 2, and 5 mg/mL) of aqueous fucoidan were prepared for the following antioxidant assays:

##### Total Antioxidant Capacity (TAC)

The total antioxidant activity of fucoidan was estimated by the phosphomolybdenum method, according to Prieto [73].

##### DPPH Radical Scavenging Activity

The activity of fucoidan at different concentrations of the free radical 1,1-diphenyl-2-picrylhydrazyl (DPPH) was measured by the Mensor method [74].

##### Hydrogen Peroxide Scavenging Activity

The antioxidant activity of fucoidan at various concentrations was studied using hydrogen peroxide by Ruch [75].

##### ABTS Scavenging Activity

The antioxidant activity of fucoidan at various concentrations was studied using the 2,2-azino-bis-3-ethylbenzthiazoline-6-sulfonic acid (ABTS) radical cation decolorization assay by Re and his colleague’s method with some modifications [76].

##### Nitric Oxide Radical Scavenging Assay

The scavenging activity of nitric oxide radicals at different fucoidan concentrations was determined by the Griess reagent [77].

##### Ferrous Ion Chelating Activity

The ferrous ion chelating activity of fucoidan at various concentrations was determined by the method of Decker and Welch [78]. In all the previous antioxidant assays, ascorbic acid (Sigma-Aldrich Co., St. Louis, MO, USA) was used as a standard substance.

##### *In Vitro* Antitumor Activity of Fucoidan

Antitumor activity was assayed for different concentrations of fucoidan obtained from *Dictyota dichotoma* var. *dichotoma*. Fucoidan powder was dissolved in phosphate buffered saline to prepare 0.05, 0.1, 0.2, 0.5, and 1 mg/mL, then sterilized using a Millipore filter (GSWP04700—MF-Millipore^®^ Membrane Filter, 0.22 µm pore size, Merck, Darmstadt, Germany) and finally preserved at 4 °C until further usage.

### 4.8. Source of Tumor Cells

Ehrlich ascites carcinoma cells (EACs) were injected intraperitoneally in Swiss albino mice (their weight was 22–25 g; their age was 70–80 days). The National Cancer Institute, Cairo University, Egypt, provided the parent cell line. This cell line was maintained in mice through serial intraperitoneal transplantation of 1 × 10^6^ viable tumor cells in 0.2 mL of saline. The tumor grows moderately rapidly, which leads to mouse death in 16 to 18 days. These cells were diluted with saline to prepare 5 × 10^6^ viable EAC cells per mL.

### 4.9. Tumor Cells Viability

Antitumor activity of different concentrations (0.05–1 mg/mL) of fucoidan obtained from *Dictyota dichotoma* var. *dichotoma* was detected by measuring tumor cell viability by the dye exclusion test (trypan blue exclusion test) according to the method of Runyang [79]. The control group included normal cells, and the final statistical results were expressed as a percentage of the control group.

### 4.10. Statistical Analysis

All determinations were carried out in triplicate, and the results are expressed as a mean ± standard deviation. The data were analyzed by one-way analysis of variance. At the same time, Duncan’s multiple comparison tests determined a significant difference between the means of triplicates using the statistical software Statistical Package for the Social Sciences (SPSS) Version 20. The IC_50_ value was calculated by concentration–response (sigmoidal fitting) with OriginPro 8.0 software.

## 5. Conclusions

In the present work, a sulfated polysaccharide “fucoidan” purified from *Dictyota dichotoma* var. *dichotoma* has been preliminarily characterized by FTIR and NMR spectra for the first time and has been tested for its antioxidant and antitumor activities using various assays. Results of the present study show that the extracted fucoidan contains fucose, glucose, galactose, mannose, xylose, and glucuronic acid. Fucoidan yield is 0.057 g/g algal dry wt, whereas its average molecular weight is 48.6 kDa. Sulfate, uronic acid, and protein contents are 83.3 ± 5.20, 22.5 ± 0.80, and 26.1 ± 1.70 mg/g fucoidan, respectively. The biological tests reveal that the extracted fucoidan is a potent antioxidant and antitumor agent. Based on these findings, future studies should be conducted to explore the mechanisms by which the obtained fucoidan induces antioxidant and antitumor activities and to facilitate the future potential application of this novel compound.

## Figures and Tables

**Table 1:** Chemical composition of fucoidan obtained from *D. dichotoma* var. *dichotoma*.

Components	mg/g Fucoidan	% Fucoidan
Sulfate	83.3 ± 5.20	8.33 ± 0.52
Uronic acid	22.5 ± 0.80	2.25 ± 0.08
Protein	26.1 ± 1.70	2.61 ± 0.17
Fucose	213.0 ± 13.0	21.3 ± 1.30
Glucose	119.0 ± 7.40	11.9 ± 0.74
Galactose	51.4 ± 6.50	5.14 ± 0.65
Mannose	27.2 ± 0.40	2.72 ± 0.04
Xylose	25.4 ± 1.90	2.54 ± 0.19
Glucuronic acid	20.9 ± 1.20	2.09 ± 0.12

Each value represents the mean ± SD of triplicate measurements.

**Table 2:** Antioxidant activity of different concentrations of fucoidan extracted from *D. dichotoma* var. *dichotoma*, compared with standard ascorbic acid.

Concentrations (mg/mL)	Ascorbic Acid	Fucoidan	Fucoidan IC_50_ (mg/mL)
	Total antioxidant capacity (TAC)		
0.1	9.16 ± 0.27	16.27 ± 0.73	1.41
0.5	16.03 ± 0.48	28.63 ± 1.29	
1	27.30 ± 1.02	45.17 ± 2.03	
2	34.06 ± 0.97	60.83 ± 2.78	
5	40.19 ± 1.21	71.76 ± 3.23	
	DPPH radical scavenging activity		
0.1	8.44 ± 0.25	15.07 ± 0.88	4.59
0.5	16.82 ± 0.27	28.25 ± 1.27	
1	17.34 ± 0.44	30.43 ± 1.37	
2	22.96 ± 0.68	39.21 ± 1.79	
5	30.63 ± 0.97	51.13 ± 2.31	
	Hydrogen peroxide scavenging activity		
0.1	5.46 ± 0.16	7.58 ± 0.34	4.84
0.5	11.07 ± 0.33	15.38 ± 0.69	
1	16.71 ± 0.50	23.29 ± 1.05	
2	22.52 ± 0.68	31.28 ± 1.42	
5	29.12 ± 0.87	40.45 ± 187	
	ABTS scavenging activity		
0.1	7.48 ± 0.22	12.27 ± 0.55	8.55
0.5	11.75 ± 0.35	19.27 ± 0.87	
1	16.56 ± 0.52	27.15 ± 1.22	
2	23.26 ± 0.70	38.13 ± 1.72	
5	30.81 ± 1.12	50.51 ± 2.27	
	Nitric oxide radical scavenging assay		
0.1	4.59 ± 0.14	9.76 ± 0.45	9.30
0.5	6.85 ± 0.21	14.57 ± 0.67	
1	9.54 ± 0.29	20.29 ± 0.91	
2	12.32 ± 0.37	26.21 ± 1.18	
5	17.92 ± 0.54	38.13 ± 1.72	
	Ferrous ion chelating activity		
0.1	1.49 ± 0.05	4.26 ± 0.19	61.9
0.5	2.57 ± 0.08	7.35 ±0.28	
1	4.10 ± 0.13	11.70 ± 0.51	
2	5.44 ± 0.13	15.53 ± 0.70	
5	6.53 ± 0.22	18.66 ± 0.84	

## Data Availability

Data will be available from the author F.W.
